# Successful Remission of Refractory Rheumatoid Pleural Effusion and Arthritis Using Sarilumab: A Case Report

**DOI:** 10.7759/cureus.74958

**Published:** 2024-12-02

**Authors:** Atsuhiko Sunaga, Takuya Inoue

**Affiliations:** 1 Department of Rheumatology, Matsushita Memorial Hospital, Moriguchi-shi, JPN

**Keywords:** biologic dmards, il-6, il-6 inhibitor, pleural effusion, pleuritis, rheumatoid arthritis, rheumatoid pleural effusion, rheumatoid pleuritis, sarilumab

## Abstract

Rheumatoid pleural effusion (RPE) is a rare complication of rheumatoid arthritis (RA) with no sufficiently established treatment. Sarilumab, a monoclonal antibody targeting the interleukin-6 receptors, is widely used to treat RA. Here, we present the case of a 68-year-old man with refractory RA and RPE, who was successfully treated with sarilumab. The patient presented with polyarthritis and pleural effusion. Based on serum and pleural fluid test results, he was diagnosed with RA and RPE but did not respond to prednisolone (PSL) therapy. Notably, arthritis and pleural effusion resolved after initiation of sarilumab treatment, with sustained remission even after PSL discontinuation. Previous reports suggest that the inflammatory mechanisms underlying pleural effusion and synovitis in RA are similar. To date, only a few studies have investigated the use of biological disease-modifying antirheumatic drugs for RPE. This case highlights the efficiency of sarilumab for RPE treatment via interleukin-6 inhibition.

## Introduction

Rheumatoid pleural effusion (RPE) is a rare complication of rheumatoid arthritis (RA), with only 3.8% of RA cases exhibiting pleural effusion on chest computed tomography (CT) scans [1]. To date, no sufficiently established treatment has been identified for RPE [2]. Moreover, reports on the use of biological disease-modifying anti-rheumatic drugs (bDMARDs) for RPE are scarce. Sarilumab is a fully human anti-interleukin (IL)-6 receptor-α monoclonal antibody that is effective against refractory RA [3,4]. Here, we present a case of refractory RA with RPE who achieved effective remission after sarilumab treatment.

## Case presentation

A 68-year-old male patient presented to our hospital with a four-month history of polyarthritis in fingers, shoulders, knees, and toes. The patient did not exhibit any respiratory symptoms or chest pain. However, he had a history of smoking 20 cigarettes daily for 20 years. Physical examination revealed tenderness and swelling in both hand joints, including multiple metacarpophalangeal and proximal interphalangeal joints. Lung and heart auscultation results were normal. Laboratory tests revealed hyperleukocytosis (12,700/μg; 68.0% with neutrophils and 14.4% with lymphocytes) and elevated erythrocyte sedimentation rate (24 mm/hour), C-reactive protein levels (0.62 mg/dL), rheumatoid factor (RF) levels (435 IU/mL), and anti-cyclic citrullinated peptide antibody levels (2,760 U/mL). All test results are presented in Table 1.

**Table 1:** Blood test result.

Components	Result	Reference range
Complete blood count		
Total leukocytes (×10^3/µL)	12.7	4.0-9.0
Neutrophils (%)	68.0	30.0-80.0
Lymphocytes (%)	14.4	15.0-60.0
Red blood cell (×10^6/µL)	4.52	4.00-5.50
Hemoglobin (g/dL)	13.5	13.7-16.8
Platelet (×10^3/µL)	335	150-420
Erythrocyte sedimentation rate (mm/h)	24	0-10
Biochemistry		
Total protein (g/dL)	6.8	6.6-8.1
Albumin (g/dL)	3.6	4.1-5.1
Blood urea nitrogen (mg/dL)	15	8.0-20.0
Creatinine (mg/dL)	0.85	0.65-1.07
Lactate dehydrogenase (U/L)	162	124-222
Blood glucose (mg/dL)	178	73-109
Serology		
C-reactive protein (mg/dL)	0.62	0.00-0.14
Matrix metalloproteinase-3 (ng/mL)	99.5	36.9-121
Rheumatoid factor (IU/mL)	435	≤15
Anti-citrullinated protein antibody (U/mL)	2760	<4.5
Anti-nuclear antibody	1:40 (homogeneous, speckled)	<1:40
T-SPOT.TB test	Negative	Negative

**Figure 1:**
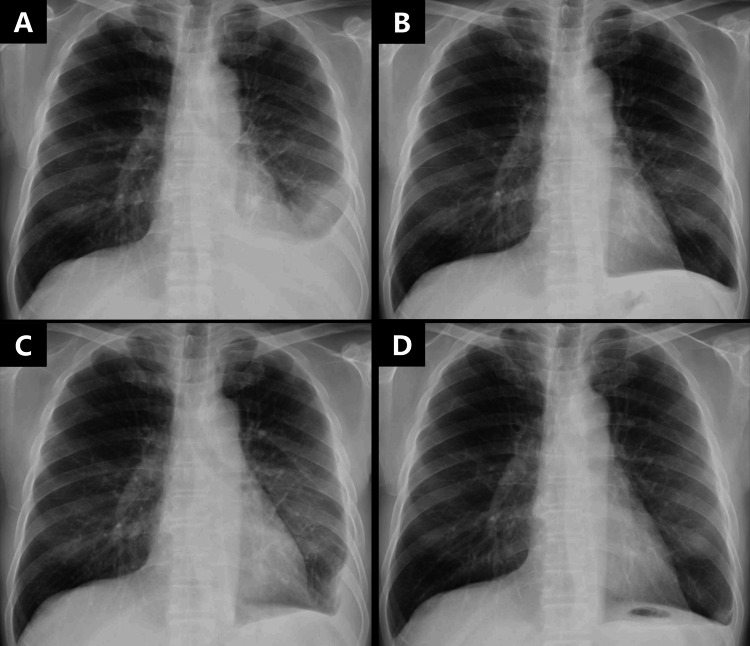
Changes in rheumatoid pleural effusion (RPE) observed via chest X-ray. (A) Left-sided RPE at initial presentation. (B) Improvement in RPE at week 7 after prednisolone initiation. (C) Relapse of RPE at week 35 after prednisolone tapering. (D) Decrease in RPE at week 6 after sarilumab initiation.

**Figure 2:**
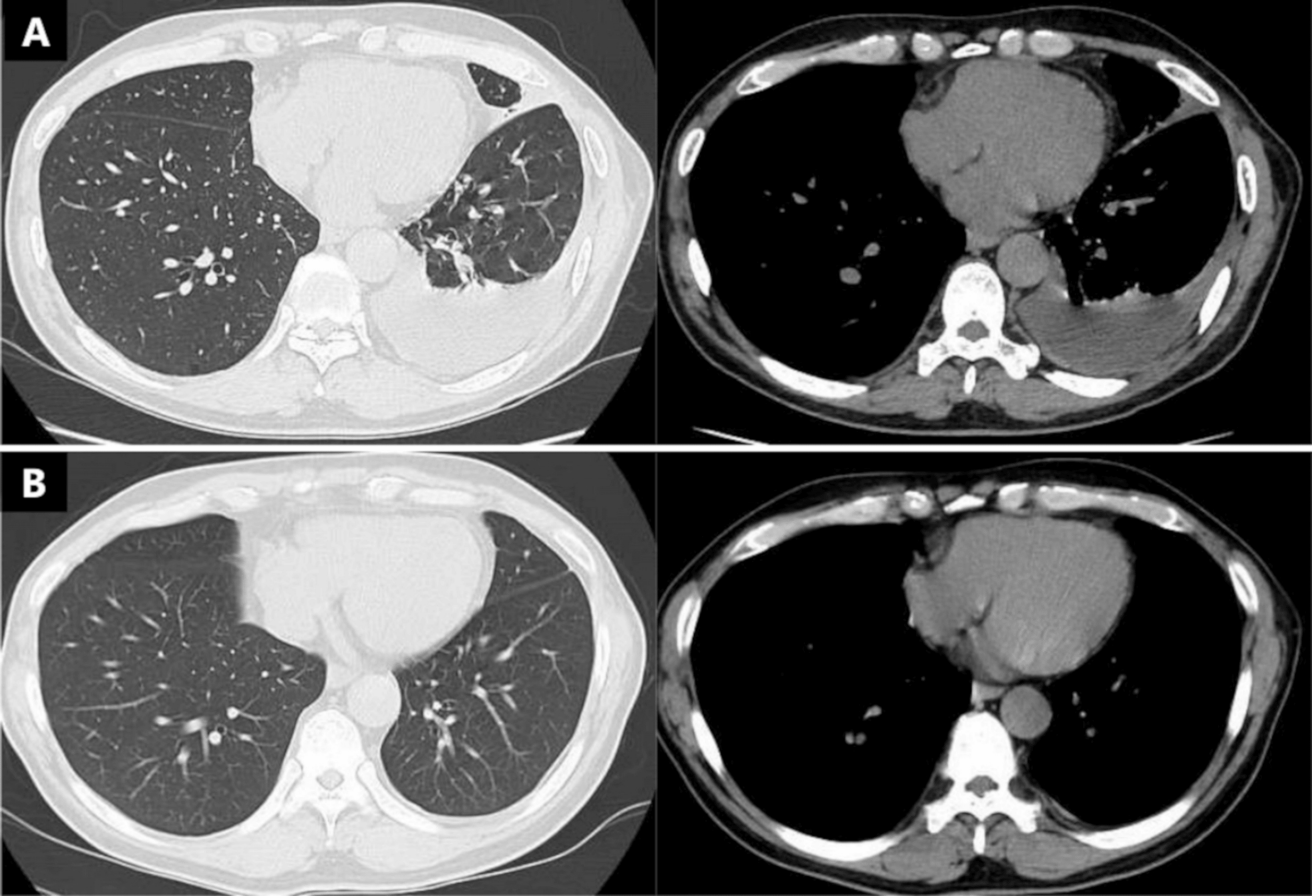
Changes in RPE observed via chest computed tomography (CT). (A) Left-sided RPE at initial presentation. (B) Resolution of RPE three months after sarilumab initiation. RPE, rheumatoid pleural effusion

Consequently, the patient was diagnosed with RA. Screening chest X-ray revealed the blunting of the left costophrenic angle and a fluid level indicating pleural effusion (Figure 1A). Chest CT scan further confirmed left pleural effusion with pleural thickening (Figure 2A). Pleural fluid analysis revealed an increased cell count with lymphocyte predominance (2,750/µL; 45.5% lymphocytes) and decreased pleural fluid pH (7.1). Biochemical analysis revealed increased total protein (4.9 g/dL) and lactate dehydrogenase (303 U/L) levels, indicating exudative pleural effusion. Compared to those in the serum, RF levels were significantly increased (716 IU/mL), adenosine deaminase levels were slightly elevated (41.6 U/L), and glucose levels were reduced (52 mg/dL) in the pleural fluid. The smear test, polymerase chain reaction, and culture revealed no common or acid-fast bacilli in the pleural fluid. All test results are presented in Table 2. Based on these findings, the patient was diagnosed with RPE.

**Table 2:** Pleural fluid test result.

Components	Result
Specific gravity	1.020
Total cell counts (×10^3/µL)	2.75
Neutrophils (%)	36.0
Lymphocytes (%)	45.5
pH	7.1
Total protein (g/dL)	4.9
Lactate dehydrogenase (U/L)	303
Glucose (mg/dL)	52
Adenosine deaminase (U/L)	41.6
Rheumatoid factor (IU/mL)	716
CEA (ng/mL)	1.5

Salazosulfapyridine (1 g/day) and prednisolone (PSL; 20 mg/day) treatment was initiated. Notably, both arthritis and pleuritis improved after seven weeks of treatment (Figure 1B). However, pleural effusion relapsed after the reduction of PSL dose and worsened with decreasing PSL doses (Figure 1C). Arthritis also flared up. The patient was subsequently administered iguratimod (50 mg/day); however, no improvement was observed. PSL dose could not be reduced below 5 mg owing to persistent pleural effusion and arthritis. At the time of treatment, methotrexate (MTX) was contraindicated in Japan; therefore, sarilumab was administered in the fifth month of treatment for disease control. Interestingly, arthritis rapidly improved, with a decrease in pleural effusion after six weeks of sarilumab treatment (Figure 1D). Additionally, the arthritis disease activity score improved (clinical disease activity index score = 5.0) and pleural effusion resolved, as confirmed by chest CT three months after initiating sarilumab (Figure 2B). Subsequently, PSL was discontinued. Effective remission of RA (clinical disease activity index score = 2.0) was achieved after eight months of sarilumab therapy, with sustained remission of both arthritis and pleural effusion observed 14 months after initiating sarilumab therapy.

## Discussion

In this study, a patient with RA and relapsing pleural effusion was successfully treated with sarilumab. Currently available treatments for RPE include non-steroidal anti-inflammatory drugs, systemic glucocorticoids (GCs), intrapleural GCs, and DMARDs [2]. However, no sufficiently established effective RPE treatment has been reported to date.

Some reports have suggested similarities in the inflammatory processes of pleural effusion, pericardial effusion, and synovitis. Common histopathological features of rheumatoid pleuritis and synovitis include the presence of rheumatoid nodules and the replacement of mesothelial cells with pseudostratified epithelioid cells of macrophage origin [1]. Pleural tissue synthesizes RFs and immunocomplexes in patients with rheumatoid pleuritis [5]. Alterations in cytokine profiles, including high tumor necrosis factor (TNF)-α, IL-6, and IL-1 levels and low interferon-γ levels, are common in both pleural effusion and RA [6,7]. These reports suggest that improving the joint disease activity resolves RPE, particularly in asymptomatic and mild cases [2]. However, before the advent of bDMARDs, RPE was not suggested to be associated with joint disease activity [8]. Collectively, pathological and cytokine profiles suggest joint disease control using bDMARDs as a beneficial treatment option for RPE.

We also reviewed previous reports on the use of bDMARDs for RPE; however, only a few cases have been documented to date. For example, Fujita et al. reported a case of GC-resistant RPE during treatment with MTX and etanercept, which was successfully treated with abatacept, MTX, and low-dose PSL [9]. Ohtsuka et al. described a case of RPE during treatment with MTX and infliximab, in which both pleural effusion and joint disease were successfully treated with tocilizumab, an IL-6 inhibitor, without GCs [10]. To date, no case of successful RPE treatment with TNF inhibitors has been reported. Instead, pleural effusion has been reported in patients treated with TNF inhibitors such as infliximab and etanercept [2,9-11]. However, TNF inhibitors cannot be considered completely ineffective for RPE treatment, as some patients exhibit improved joint disease activity after TNF inhibitor therapy. Our case treated with sarilumab and a previous case treated with tocilizumab [10] suggest the potential of IL-6 inhibitors to control both pleural effusion and joint activity, even without GCs. Additionally, in our case, pleural effusion improved rapidly after six weeks of sarilumab initiation. Sarilumab has been reported to exhibit higher IL-6 receptor binding affinity and potency than tocilizumab in RA [12]. Consistently, this case showed the rapid and potent efficacy of sarilumab for RPE and arthritis.

## Conclusions

RPE is a rare complication of RA with no effective treatment. Moreover, studies on the use of bDMARDs for RPE treatment are scarce. Previous studies have suggested similarities in the inflammatory processes of pleuritis and synovitis in RA. This case suggests that IL-6 inhibitors effectively induce the remission of refractory RPE, even without GCs. Furthermore, our findings highlight sarilumab as an effective fast-acting therapeutic for both RPE and arthritis.
